# Segmentation of *C. elegans* germline nuclei

**DOI:** 10.17912/micropub.biology.001062

**Published:** 2023-12-11

**Authors:** Cristina Piñeiro López, Ana Rita Rodrigues Neves, Ivana Čavka, Oane Jan Gros, Simone Köhler

**Affiliations:** 1 Cell Biology and Biophysics, European Molecular Biology Laboratory, Heidelberg, Baden-Württemberg, Germany; 2 Collaboration for joint PhD degree between EMBL and Heidelberg University, Faculty of Biosciences, Heidelberg, Germany

## Abstract

Immunofluorescence microscopy is a widely adopted method for studying meiotic prophase in the nematode model organism, *Caenorhabditis elegans* . An in-depth examination of specific meiotic processes requires the quantitative analysis of immunofluorescence images, which often involves the segmentation of individual cells or nuclei. Here, we introduce our image analysis pipeline to automate significant portions of this task. This pipeline relies on the powerful deep learning model Cellpose 2.0 to segment cellular structures. To further improve the segmentation accuracy for germline nuclei stained for chromatin or synaptonemal complexes, we retrained the generalist Cellpose model and integrated our data processing pipeline into the easy-to-use Cell-ACDC image analysis software. Our pipeline thus makes deep learning-based segmentation of nuclei in the distal germline of *C. elegans* accessible for users without coding experience.

**Figure 1. Segmentation of germline nuclei in C. elegans using Cell-ACDC and Cellpose:**
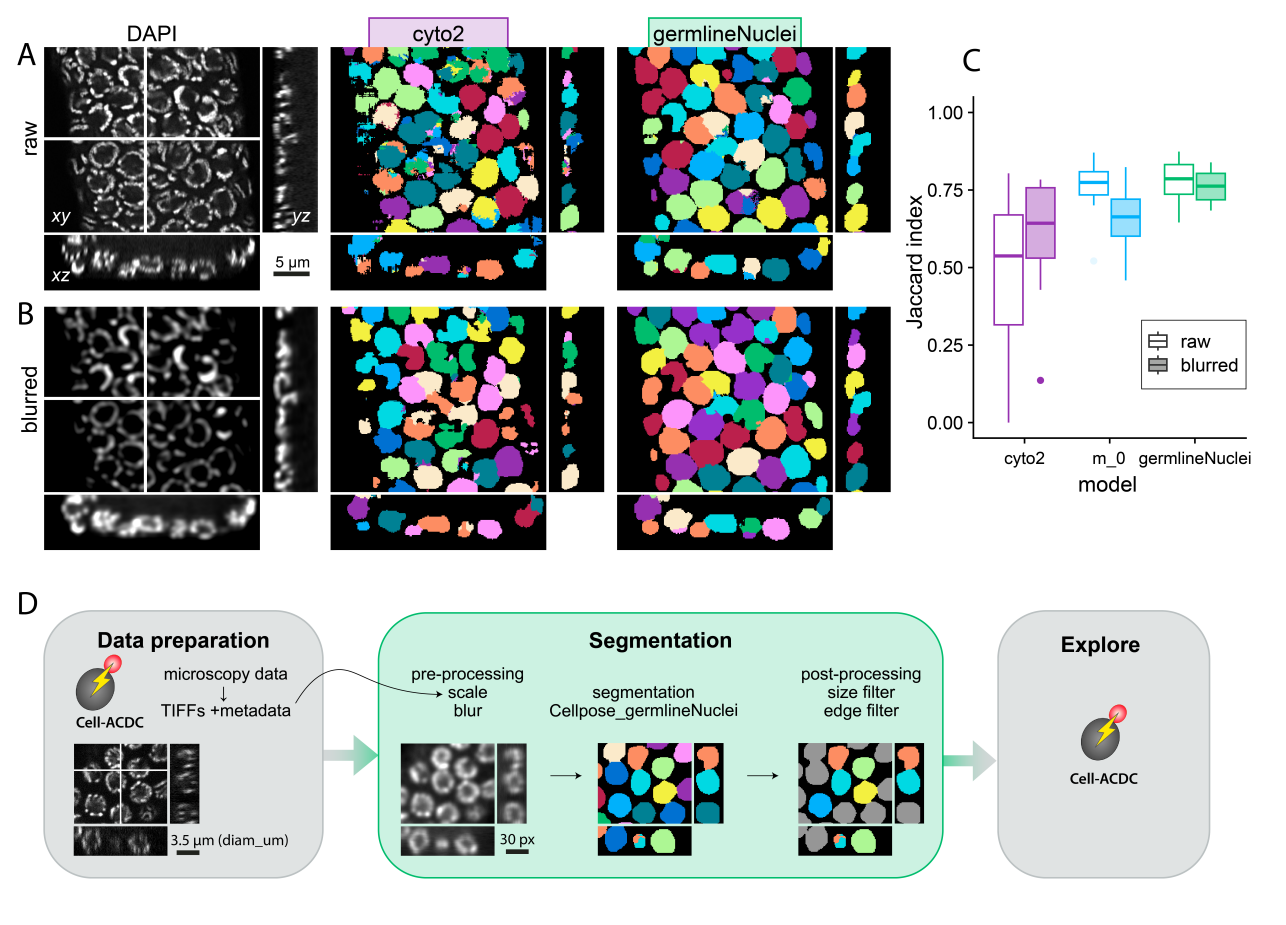
(A) Orthogonal views of a cropped z-stack of DAPI-stained *C. elegans* distal germline nuclei (left, imaged with AiryScan 880 microscope) were segmented with the cyto2 model of Cellpose (cyto2, purple, center) or a retrained model using 108 manually segmented "training" images (germlineNuclei, green, right). The fragmentation of individual nuclei is reduced with the custom model. White lines indicate *x* and *y* positions shown in the *xz* and *yz* orthogonal views. (B) Blurring by a factor of 2.5 (left: orthogonal views of blurred DAPI images) further improves the segmentation outcome for the cyto2 (center) and the custom germlineNuclei model (right). (C) The success of training is assessed by calculating the Jaccard index (Stringer et al., 2021; Pachitariu & Stringer, 2022) for 12 manually segmented "test" images. Jaccard indices for raw images are shown with open box plots, and with filled box plots for blurred images. Boxes show the interquartile range, medians are indicated by horizontal lines. Whiskers show smallest and largest value within 1.5 times the interquartile range below the 25th or above the 75th percentile, respectively. Blurring the data improves the Jaccard index for the cyto2 model (purple). By contrast, a re-trained custom model (m_0, blue) improves the segmentation of raw data but the Jaccard index is similar to the original cyto2 model for blurred data. Augmenting the training data improves the segmentation accuracy for both raw and blurred data sets (germlineNuclei, green). (D) The segmentation pipeline including all pre- and post-processing steps was implemented in Cell-ACDC (Padovani et al., 2022). Images shown are DAPI-stained *C. elegans* germline nuclei obtained with a SoRA spinning disk microscope.

## Description

During meiotic prophase I, homologous chromosomes must pair, synapse, and recombine to ensure the correct segregation of homologs in the first meiotic division. The *C. elegans* germline offers unique advantages to study these processes since this tissue contains about 1000 nuclei which are organized in a temporo-spatial manner. This organization allows assessing the progression through all meiotic prophase I stages in single animals (Kimble & White, 1981; Hillers et al., 2017) . However, the quantification of individual processes often requires labor-intensive and bias-prone manual quantification of specific markers in immunofluorescence images. To make this task more robust and allow for higher throughput, automated image quantification techniques are necessary. To this end, the nuclei must first be segmented into independent objects or masks to automate image quantification. A recent publication demonstrated for the first time a semi-automated image analysis pipeline for the segmentation and quantification of *C. elegans* meiotic nuclei (Toraason et al., 2021) . However, this pipeline relies on the use of proprietary software, limiting the use of this tool. At the same time, recent developments in deep learning have greatly improved image segmentation tasks. In particular, pre-trained models using convolutional neural networks such as Cellpose (Stringer et al., 2021; Pachitariu & Stringer, 2022) or StarDist (Schmidt et al., 2018; Weigert & Schmidt, 2022) have significantly simplified the implementation of deep learning-based approaches by non-experts.

In this micropublication, we share our re-trained Cellpose 2.0 model that robustly recognizes distal germline nuclei across three different imaging platforms. We implemented the full pipeline in Cell-ACDC (Padovani et al., 2022) , which provides an easy-to-use graphical user interface that also simplifies manual quality control and correction of segmentation results.

Generally, segmenting germline nuclei is challenging since the chromatin staining of partially condensed and paired chromosomes is discontinuous. However, nuclei are easily recognizable by eye for their round shape ( Fig. 1A ). We use Cellpose as it has a strong bias for similar round objects (Stringer et al., 2021) . Indeed, the pre-trained "cyto2" Cellpose model already segments many germline nuclei correctly without additional training. However, other nuclei were split into multiple parts ( Fig. 1A, center ). We therefore blurred the data prior to segmentation which reduces the fragmentation. Still, the pre-trained model results in a noticeable amount of segmentation errors ( Fig. 1B, center ).

To further improve the segmentation accuracy, we re-trained Cellpose with a set of 108 training images of nuclei from distal *C. elegans* germlines (Pachitariu & Stringer, 2022) . Cellpose reconstructs 3D segmentations from 2D predictions of orthogonal views ( *xy* , *xz* , and *yz* slices, respectively) (Stringer et al., 2021) . Thus, we generated ground truth data of 2D orthogonal views of 3D data. Annotating 2D orthogonal views reduces the amount of ground truth data required since only a small number of images must be annotated manually rather than entire *z* -stacks. To generalize our model for different imaging modalities, we generated ground truth data for fluorescence micrographs of chromatin (DAPI staining) or synaptonemal complex staining from different imaging modalities (AiryScan, spinning disk confocal microscope with and without SoRA disc, DeltaVision). Since Cellpose targets objects with a diameter of about 30 pixels (Stringer et al., 2021) , we rescaled all training data to a pixel size of 0.117 µm/pixel, which converts the estimated diameter of a nucleus from 3.5 µm to 30 pixels.

Our re-training improved the Jaccard index from 0.47±0.24 ( Fig. 1C, cyto 2 model, purple open box plots) to 0.76±0.09 ( Fig. 1C, m _0, blue open box plots). However, many nuclei were still not segmented correctly and blurring the data did not improve the accuracy. The Jaccard indices of both the "cyto2" model ( Fig. 1C, purple filled box plots ) and the retrained "m_0" model ( Fig. 1C, blue filled box plots ) are similar with values of 0.61±0.18 and 0.66±0.11, respectively. To further improve the segmentation of germline nuclei without the need for generating more ground truth data, we performed basic geometric transformations (rotation, flipping) and data blurring on the training data. This training data augmentation further improved the Jaccard index to 0.78±0.07 without blurring and 0.76±0.05 with blurring, respectively ( Fig. 1C, germlineNuclei model, green ).

Indeed, this "germlineNuclei" model generated with augmented training data was able to segment most nuclei in images of distal germlines correctly although some errors still occur in challenging data sets ( Fig. 1A -B, right).

To facilitate the use of our segmentation pipeline, we integrated this custom model in Cell-ACDC (Padovani et al., 2022) ( Fig. 1D ). This software imports microscopy data and resaves the data into easy-to-use tiff-stacks along with its metadata. We developed a plugin that allows for the segmentation of germline nuclei using these re-saved tiff-stacks. In the graphical user interface, the user inputs their expected nucleus diameter in µm, while the pixel size of the original microscopy image is pre-populated automatically from the metadata. This information is used to rescale the microscopy images to a nucleus diameter of 30 pixels, and to calculate the anisotropy in the microscopy dataset. A user-specified blur factor will be used to blur the data before segmentation by Cellpose with our custom model. To remove incomplete nuclei, the parameter " *clean_borders* " may be set to true, which will remove segmentation masks touching the borders of the image stack.

Furthermore, Cell-ACDC includes post-processing steps to filter masks according to their size, solidity, elongation, or thickness in *z* (Padovani et al., 2022) . Cell-ACDC also allows the user to visualize and correct the segmentation as needed. This segmentation pipeline for germline nuclei is available by selecting the "Cellpose_germlineNuclei" model within the segmentation module of Cell-ACDC (see methods and Padovani et al., 2022). We anticipate that this image segmentation will facilitate the use of (semi-)automated image quantification pipelines in the field.

## Methods

*Image acquisition*

To obtain training data, *C. elegans* germlines were dissected and stained with DAPI as previously described (Phillips et al., 2009; Köhler et al., 2017). Germlines were mounted in ProLong Diamond or ProLong Glass. For images of synaptonemal complexes, living *C. elegans* animals expressing fluorescent synaptonemal complex markers were mounted on agarose pads (Rivera Gomez & Schvarzstein, 2018) . Z-stack images were acquired on a Zeiss AiryScan 880 (63x oil immersion objective, NA 1.4) in fast mode, Zeiss AiryScan 980 (63x oil immersion objective, NA 1.4) in 4xSR mode, an Olympus Spinning Disk microscope (60x oil immersion objective, NA 1.42; or 100x silicone oil immersion objective, NA 1.35) with a 50 µm disk or a SoRa disk and 3.2x magnification, or a GE DeltaVision Elite microscope (100x oil immersion objective, NA 1.4). AiryScan images were processed with 3D default (880) or 'low' (980) settings in ZenBlack (880) or ZenBlue (980) software, and DeltaVision images were iteratively convolved using the SoftWoRx package. We selected and cropped 70 *xy* , 26 *xz* , and 24 *yz* slices using Fiji (Schindelin et al., 2012) to obtain 2D orthogonal views from z-stacks.

*Training Cellpose model*

Ground truth masks for the 2D orthogonal views were annotated by one of four different experts using the Cellpose 1.0 or 2.0 graphical user interface (Stringer et al., 2021; Pachitariu & Stringer, 2022) . Ground truth data (120 images) was then randomly split into training (108 images) and test data (12 images) using the *scikit-learn 1.3.0* library. All data was rescaled to 0.117 µm/pixel. The *cytotorch2_0* model (cyto2) was re-trained using Cellpose 2.0 and the following parameters: n_epochs=500, momentum=0.9, weight_decay=1e-05, learning_rate=0.05, min_train_masks=2, normalize=True, and rescale=True (Pachitariu & Stringer, 2022) . To improve training efficiency, we also augmented the training data set by random mirroring (horizontal and/or vertical), random rotations (0-90 degrees), adding noise, rescaling the intensity, and/or blurring the data (0-3.5 blur factor) using the *scikit-image* 0.21.0, *SciPy* 1.10.1 and *openCV* 4.8.0 libraries. All ground truth data and a jupyter notebook containing the training script are available at https://github.com/KoehlerLab/Cellpose_germlineNuclei . Note that the segmentation accuracy using our shared custom model will depend on the similarity of the data to our training data. Therefore, re-training with additional and user-specific ground truth data may be necessary to obtain the desired segmentation accuracy.

*Cell-ACDC*

Cell-ACDC software (v1.4.16; Padovani et al., 2022), and its user manual are available at https://github.com/SchmollerLab/Cell_ACDC . Cell-ACDC is first used to load and (optionally) pre-process the data (Data preparation in Fig. 1D ). This step will also load and save the image metadata. Then, the segmentation module (Segmentation in Fig. 1D ) is launched selecting the "Cellpose_germlineNuclei" model. Selecting this option will automatically download the necessary files for our custom segmentation pipeline. Alternatively, users can also install the model manually (see Padovani et al., 2022). Selecting the "Cellpose_germlineNuclei" model in the Cell-ACDC segmentation module will initiate a custom plugin containing all pre- and post-processing steps (rescaling nuclei of given diameter to 30 pixels, blurring data, removing masks touching the borders of the image stack) and the Cellpose segmentation using our custom Cellpose model. The plugin will rescale the data to convert an object of *diameter_um* (user-specified diameter of the nuclei in µm; default is 3.5 µm) to 30-pixel diameter using *SciPy 1.11.1* . The rescaled image will then be blurred by *blurfactor* (default 2.5 for chromatin staining; 3.5 is recommended for deconvolved images or synaptonemal complex staining) using the *SciPy 1.11.1* library. Images are then segmented in 3D with Cellpose 2.0 (Stringer et al., 2021; Pachitariu & Stringer, 2022) . While the anisotropy parameter for 3D Cellpose segmentation is calculated automatically from the image metadata, the user can adjust the *cellprob_threshold* (we use the default value of 0.0). To remove potential incomplete nuclei from the segmented results, activating the *clean_borders* parameter will delete any masks that touch the first or last slice in *z* , or that are within 2 pixels from the edges of the stack in x or y. A more detailed instruction on how to use the "Cellpose_germlineNuclei" is given on our GitHub page.

## Extended Data

Description: python scripts, training data, and custom Cellpose model for Cellpose_germlineNuclei. Resource Type: Software. DOI: 10.22002/3yvt7-dm257
